# Genetic loci associated with skin pigmentation in African Americans and their effects on vitamin D deficiency

**DOI:** 10.1371/journal.pgen.1009319

**Published:** 2021-02-18

**Authors:** Ken Batai, Zuxi Cui, Amit Arora, Ebony Shah-Williams, Wenndy Hernandez, Maria Ruden, Courtney M. P. Hollowell, Stanley E. Hooker, Madhavi Bathina, Adam B. Murphy, Carolina Bonilla, Rick A. Kittles

**Affiliations:** 1 Department of Urology, University of Arizona, Tucson, Arizona, United States of America; 2 Department of Population and Quantitative Health Sciences, Case Western Reserve University, Cleveland, Ohio, United States of America; 3 Department of Epidemiology and Biostatistics, University of Arizona, Tucson, Arizona, United States of America; 4 Department of Medical and Molecular Genetics, Indiana University, Indianapolis, Indiana United States of America; 5 Department of Medicine, University of Chicago, Chicago, Illinois, United States of America; 6 Department of Surgery, Cook County Health and Hospitals System, Chicago, Illinois, United States of America; 7 Division of Health Equities, Department of Population Sciences, City of Hope Comprehensive Cancer Center, Duarte, California, United States of America; 8 Department of Urology, Northwestern University, Chicago, Illinois, United States of America; 9 Departamento de Medicina Preventiva, Faculdade de Medicina, Universidade de São Paulo, São Paulo, Brazil; 10 Population Health Sciences, Bristol Medical School, University of Bristol, Bristol, United Kingdom; Stanford University School of Medicine, UNITED STATES

## Abstract

A recent genome-wide association study (GWAS) in African descent populations identified novel loci associated with skin pigmentation. However, how genomic variations affect skin pigmentation and how these skin pigmentation gene variants affect serum 25(OH) vitamin D variation has not been explored in African Americans (AAs). In order to further understand genetic factors that affect human skin pigmentation and serum 25(OH)D variation, we performed a GWAS for skin pigmentation with 395 AAs and a replication study with 681 AAs. Then, we tested if the identified variants are associated with serum 25(OH) D concentrations in a subset of AAs (n = 591). Skin pigmentation, Melanin Index (M-Index), was measured using a narrow-band reflectometer. Multiple regression analysis was performed to identify variants associated with M-Index and to assess their role in serum 25(OH)D variation adjusting for population stratification and relevant confounding variables. A variant near the *SLC24A5* gene (rs2675345) showed the strongest signal of association with M-Index (*P* = 4.0 x 10^−30^ in the pooled dataset). Variants in *SLC24A5*, *SLC45A2* and *OCA2* together account for a large proportion of skin pigmentation variance (11%). The effects of these variants on M-Index was modified by sex (*P* for interaction = 0.009). However, West African Ancestry (WAA) also accounts for a large proportion of M-Index variance (23%). M-Index also varies among AAs with high WAA and high Genetic Score calculated from top variants associated with M-Index, suggesting that other unknown genomic factors related to WAA are likely contributing to skin pigmentation variation. M-Index was not associated with serum 25(OH)D concentrations, but the Genetic Score was significantly associated with vitamin D deficiency (serum 25(OH)D levels less than 12 ng/mL) (OR, 1.30; 95% CI, 1.04–1.64). The findings support the hypothesis suggesting that skin pigmentation evolved responding to increased demand for subcutaneous vitamin D synthesis in high latitude environments.

## Author summary

Genome-wide association and replication study for skin pigmentation was performed in African Americans, and then the implication of the skin pigmentation genes in serum vitamin D variation was assessed. A variant, rs2675345, near *SLC24A5* showed the strongest associations with skin pigmentation. A Genetic Score calculated using the top variants from 3 genomic regions, *SLC24A5*, *SLC45A2* and *OCA2*, and West African genomic ancestry together account for a large proportion of skin pigmentation variation. The pattern of association between the Genetic Score and skin pigmentation was different between men and women suggesting an interaction between sex and genetic variation. The Genetic Score from the same 3 skin pigmentation gene variants was also associated with severe vitamin D deficiency, defined as serum 25(OH)D levels <12 ng/mL. However, skin pigmentation and vitamin D pathway gene variants account for a small but significant proportion of serum vitamin D variation. The results suggest that genomic variations strongly control skin pigmentation and also influence serum vitamin D levels.

## Introduction

A number of studies have identified single nucleotide polymorphisms (SNPs) associated with pigmentation traits, such as skin, eye, and hair color, sensitivity to sun or tanning, and freckles, using genome-wide approaches [1–7]. Recent studies focusing on skin pigmentation variation in African populations identified novel loci associated with skin pigmentation [8,9]. Another study in Latin American countries identified additional novel loci [7]. A GWAS meta-analysis of skin pigmentation in admixed populations did not identify a novel variant, but validated some of the major findings from previous studies in African and admixed populations [10]. These studies suggest that population-specific variants affecting skin pigmentation variation exist. However, there has been no genome-wide association study (GWAS) of skin pigmentation aiming to understand the genetic variation for skin pigmentation in African Americans (AAs). Pigmentation traits are complex phenotypes that show great variation across human populations. There is evidence suggesting that such variation has been shaped by natural selection at different latitudes, to prevent DNA damage by ultraviolet radiation to the skin and/or to guarantee enough synthesis of vitamin D, given that vitamin D synthesis is initiated in the skin [11]. Sexual selection has also been implicated as an important factor in the evolution of skin pigmentation and may explain the observed difference in skin color between males and females [12–15].

The importance of elucidating the genetic basis of pigmentation traits expands beyond a better understanding of the evolutionary mechanisms that shaped some of the most visible phenotypic traits. It will also provide a better understanding of genetic risk factors for skin cancer [16–19] and vitamin D deficiency in different populations [11,20]. While many studies have explored the relationship between skin pigmentation gene variants and skin cancer risk, there is paucity of studies aiming to understand the relationship between skin pigmentation gene variants and vitamin D deficiency in AAs who are disproportionately affected by vitamin D deficiency. Identifying genetic variants that affect pigmentation traits is also important for forensic science [21,22]. Furthermore, our understanding of the genetic basis of skin pigmentation may have social implications given its conspicuous nature [23–26].

In order to further understand genetic factors that affect human skin pigmentation, we performed a GWAS in AAs. Then, we assessed the effects of skin pigmentation variants on serum 25-hydroxyvitamin D [25(OH)D] variation and vitamin D deficiency [serum 25(OH)D levels <12 ng/mL] in male AAs who were part of a study on serum 25(OH)D. Instead of assessing relative homogeneous populations with respect to skin pigmentation, we studied AAs, as it is well documented that this population exhibits a vast range of variation with respect to skin pigmentation and genetic ancestral proportions [27,28].

## Materials and methods

### Ethics statement

Written consent was obtained from all study subjects. This study was approved by the Institutional Review Boards at Howard University (IRB-99-MED-20) and Northwestern University (STU00005398).

### Study participants

Study participants in the discovery dataset were recruited in Washington, D.C. and Chicago, IL. Participants in the replication set were recruited in Washington, D.C., Cincinnati, OH, and Chicago, IL. The participants from Washington, D.C. were recruited at Howard University for one of two studies on human pigmentation [29] or serum vitamin D levels and prostate cancer in AAs [30]. The participants from Chicago were recruited for a vitamin D and prostate cancer study at five hospitals as well as from community health events [31,32]. The participants from Cincinnati were recruited as a part of a study to investigate the relationship between self-reported race, genetic ancestry, socio-economic status, and skin color [33]. All of the study participants were self-identified AAs. After receiving consent, study coordinators administered a questionnaire and obtained demographic information. Blood samples for DNA analysis and serum 25(OH)D assays were collected at the time of recruitment.

### Skin pigmentation measurements

Constitutive skin pigmentation in the sun-protected area of skin on the inner upper arm was measured by trained study coordinators or research assistants using a computerized portable narrow-band reflectometer, called DermaSpectrometer (Cyberderm, PA) [34,35]. The DermaSpectrometer output is expressed in terms of erythema (E) and melanin (M) indices from 0–100%, where higher M values denote higher pigment content. Three separate measurements were recorded for one arm and the average of M was used in all the analyses.

### Serum vitamin D measurements

Serum 25(OH)D were measured previously [31], and in the current study, we included 734 individuals with skin color measurements. The serum samples were stored at −20°C until the 25(OH)D assay was performed. Total 25(OH)D concentration was measured using the Diasorin chemiluminescence immunoassay method in the Department of Pathology NorthShore University HealthSystem. In this study, we considered individuals with serum 25(OH)D levels <12 ng/mL as severely vitamin D deficient based on the Institute of Medicine dietary reference for vitamin D intake [36] as well as an Endocrine Society Clinical Practice Guideline [37,38].

### Genotyping

The discovery set (n = 395) was genotyped using two different genome-wide SNP arrays. Washington, D.C. samples were genotyped using the Illumina 1M array as part of a GWAS for prostate cancer in African descent populations (n = 215) [39]. Additional samples (n = 180) from the Washington D.C. pigmentation gene study and Chicago vitamin D study were genotyped using the Affymetrix PanAFR array for this study. Following standard GWAS quality control (QC) recommendations, SNPs with low genotyping call rate (<95%), Hardy-Weinberg Equilibrium (HWE) *P*< 0.001, and minor allele frequency (MAF) <1% were removed. Poorly genotyped samples (<95%) and related individuals (Identity-by-descent PI-HAT >0.2) were also removed. After QC, both GWAS datasets were merged and principal component analysis was performed to assess if there were systematic differences between them. We did not find evidence of systematic error. Genotyping to replicate the findings from the discovery cohort was performed using the Agena Bioscience MassARRAY iPLEX platform (n = 681). The pooled dataset consists of a total of 1,076 AAs.

We also selected 24 previously identified SNPs associated with pigmentation traits in European populations and admixed populations for replication. Some of these SNPs were not successfully imputed in the Affymetrix dataset, so genotyping was performed for the Affymetrix dataset using the MassARRAY platform, resulting in a total of 1,066 participants in this replication analysis after QC. Genotyping of 38 SNPs in 8 vitamin D metabolic and signaling pathway genes was performed in our previously study [31], and SNP selection criteria was described previously [40]. These include previously GWAS identified SNPs that were associated with serum vitamin D levels in European populations [41,42]. To estimate genetic ancestry, a validated set of ancestry informative markers to estimate continental ancestry information in admixed populations [43] were genotyped for all the samples including three parental population sets. The parental populations used to estimate admixture proportions included 243 Europeans (from England, Germany, Ireland and Spain), 279 West Africans (from Cameroon, Nigeria and Sierra Leone), and 214 Native Americans (Cheyenne, Maya, Pima, Pueblo, and Mayans).

### Imputation

Genome lift-over was performed for the Illumina dataset to map the SNPs using the Human Genome version 19 (hg19). Shapeit.v2 was used for phasing genotype data [44]. Imputation was performed using Impute v2.3.2 and 1000 Genome Project as the reference panel [45,46]. After the imputation, variants with Infor Score <0.5 were removed. Before imputation, there were 1,013,952 SNPs in the Illumina dataset and 1,714,384 SNPs in the Affymetrix dataset. After imputation, there were 11,065,735 SNPs in the Illumina dataset and 9,218,475 SNPs in the Affymetrix dataset. We pooled both datasets and removed variants with a genotype missing rate > 5%, HWE *P*<1.0 x 10^−5^, and MAF < 1%. A total of 7,169,107 SNPs were used for subsequent analyses.

### Statistical analysis

M-Index was log-transformed to normalize the distribution in the population. For the analysis of M-Index in the GWAS discovery dataset, a linear model was used adjusting for age, sex, and the first 3 principal components (PCs). The model building process included up to 10 PCs in the regression model. The final model includes minimum number of PCs necessary to correct for population structure and reduce genomic inflation. In the replication and pooled analysis, West African Ancestry (WAA) was used instead of principal components. Individual admixture proportion was estimated using STRUCTURE v2.3, a model-based clustering method [47,48]. STRUCTURE was run under the admixture model using *K* = 3 ancestral populations (West African, European, and Native American). We used a burn-in length of 100,000 for 100,000 repetitions. For the GWAS analyses we used *P*<5.0 x 10^−8^ as the genome-wide significant threshold and *P*<0.05 as a statistically significant cutoff for replication.

A weighted Genetic Score was calculated using the top 3 and 10 associated SNPs for skin pigmentation. The weighted Genetic Score is sum of the effects of each SNP weighted by its estimated effect size (*β*) from regression model, $Genetic\;Score=\sum_{j=1}^{m}\left(\chi_{ij}\beta_{j}\right)$, where *m* is number of SNP included and χ_*ij*_ is the genotype for the *i*^*th*^ individual and *j*^*th*^ SNP (coded as 0, 1, and 2 for increase number of allele associated with darker skin pigmentation) [49]. One SNP from a single genomic region was included for calculation. When there were more than 2 SNPs with *P*<0.05 in a same genomic region, the SNP with the lowest *P*-value was used after conditional analysis to test if the SNP with the second lowest *P*-value was independently associated with M-Index by including the lead SNP in the region in the regression model. The Genetic Score calculated from top SNPs from 3 and 10 loci were initially assessed to estimate variance in skin pigmentation. Because the top 3 and top 10 SNPs accounted for a similar amount of skin pigmentation variation, subsequently, we focused our analysis using the Genetic Score estimated from the top 3 SNPs. Sex-specific Genetic Scores were also calculated. First, linear regression analysis was performed separately for males and females for the top 3 SNPs associated with M-Index in the pooled dataset. Then, *β* coefficients obtained for each SNP in males and females were used for calculation of sex-specific Genetic Scores.

Associations with serum 25(OH)D levels were tested using linear regression models adjusting for age, UV season (season of blood draw), total vitamin D intake, recruitment site, and WAA as described previously [31]. Log-transformed serum 25(OH)D levels were used in the linear regression analysis. Genetic Score from vitamin D metabolic and signaling pathway gene variants were calculated using the same formula described above using the SNPs associated with serum 25(OH)D levels in our dataset. Binary logistic regression was used to examine the association between skin pigmentation gene variants and vitamin D deficiency adjusting for age, UV season, total vitamin D intake, recruitment site, WAA, and also Genetic Score calculated from vitamin D metabolic pathway gene variants associated with serum 25(OH)D levels. Statistical analysis was performed using PLINK 1.07 [50], SPSS (IBM Corp., Armonk, NY), and R.

## Results

### Study participants’ characteristics

Because the majority of study participants came from prostate cancer studies, our study participants tend to be older (mean age of 57.2 in the discovery and 51.4 in the replication dataset, **Table 1**). Men were also over-represented in both datasets (90.9% and 85.9% male in the discovery and replication dataset, respectively). The discovery dataset had older and more male study participants. Mean M-Index and WAA were similar in the discovery and replication datasets. Serum 25(OH)D measurements were available for 734 men in the discovery and replication datasets. Mean serum 25(OH)D concentration was 19.5 ng/mL, and over 50% of participants had 25(OH)D levels that were categorized as deficient to severely deficient.

**Table 1:** Characteristics of study participants.

	Discovery (n = 395)	Replication (n = 681)	*P*^1^	Participants with serum vitamin D data (n = 734)
Age, mean (SD)	57.2 (15.0)	51.4 (14.6)	<0.001	60.3 (9.5)
Sex, n (%)			0.008	
Male	359 (90.9%)	585 (85.9%)		734 (100)
Female	36 (9.1%)	96 (14.1%)		0 (0)
M-Index, mean (SD)	52.6 (10.0)	52.2 (9.4)	0.72	52.7 (9.6)
West African Ancestry, mean (SD)	79.6 (14.7)	78.7 (12.7)	0.24	79.0 (12.6)
Serum 25(OH)D levels ng/mL, mean (SD)				19.5 (10.0)
Vitamin D status^2^, n (%)				
Severely deficient (<12 ng/mL)				180 (24.5)
Deficient (≥12 ng/mL, <20 ng/mL)				232 (31.6)
Insufficient (≥ 20 ng/mL, < 30 ng/mL)				214 (29.2)
Sufficient (≥30 ng/mL)				108 (14.7)

^1^ *P*-values from comparison between discovery and replication datasets.

^2^ Based on the Endocrine Society Clinical Practice Guideline (Holick, 2007; Holick et al. 2011)

### Variants associated with skin pigmentation

**Fig 1:**
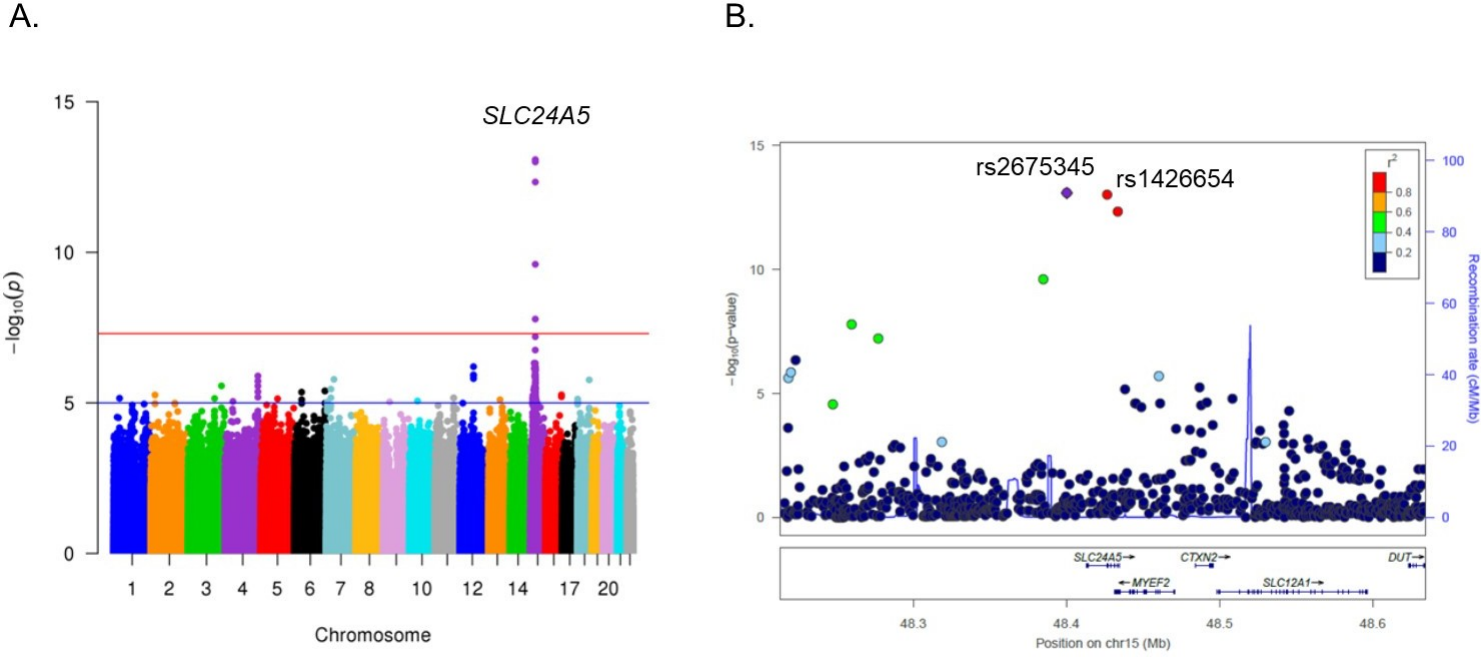
GWAS results: Manhattan plot showing a strong association between *SLC24A5* SNPs on chromosome 15 and M-Index (**A**) and LocusZoom plot of *SLC24A5* region (**B**).

One locus reached genome-wide significance in the discovery dataset (**Fig 1A and 1B**), and a SNP, rs2675345 near the *SLC24A5* gene on chromosome 15, showed the strongest signal of association (*P* = 8.4 x 10^−14^). The previously identified SNP, rs1426654, was the second most significantly associated SNP (*P* = 9.9 x 10^−14^) [51,52]. These two SNPs were strongly linked with *r*^*2*^ = 0.97. We did not find evidence of genomic inflation (**S1 Fig**), and inclusion of additional PCs did not change the analysis results. The second signal of association was found on the *TRHDE* (thyrotropin-releasing hormone degrading enzyme) gene on chromosome 12 (**S2 Fig**). The SNP, rs11179301, showed the strongest association in the region (*P* = 6.28 x 10^−7^).

Associations between skin pigmentation and 4 previously identified loci in African populations [8,9] were explored. Index SNPs from these four loci (*SNX13*, *SMARCA2/VLDLR*, *TMEM138/DDB1*, and *MFSD12*) were not successfully genotyped or imputed in our study. SNP rs2093835 was the second most strongly associated SNP in the *SMARCA2/VLDLR* region in African populations [9], but this SNP was not significantly associated with M-Index (*P* = 0.59) in our AA samples. These 4 genomic regions were further explored to identify variants associated with M-Index. However, these 4 regions did not show strong evidence of association with skin pigmentation, and we did not find any variant with *P*< 1.0 x 10^−3^ (**S3 Fig**).

Seventeen SNPs with *P*-value less than 1.0 x 10^−5^ from 15 genomic regions were selected for replication analysis (**Table 2**). Two SNPs, rs2675345 near *SLC24A5* on chromosome 15, and rs12644472 in the *ATP8A1/ GRXCR1* region on chromosome 4, were replicated with *P*<0.05 and *Beta* coefficient in the same direction. The *SLC24A5* SNP, rs2675345, was strongly associated with M-Index and in the pooled dataset, it had a *P*-value of 4.0 x 10^−30^. The *ATP8A1/GRXCR1* region SNP, rs12644472, was replicated with *P* = 0.03. In the pooled dataset, the association between rs12644472 and M-Index did not reach genome-wide significance (*P* = 2.1x10^-5^). Three SNPs in the *TRHDE* gene were selected for the replication study. These SNPs were strongly linked to each other (*r*^*2*^>0.8), and they were not associated with M-Index in our replication dataset. Given strong suggestive associations with M-Index in the discovery cohort and a potential biological significance, we further explored the *TRHDE* gene by genotyping additional SNPs in our replication cohort, and rs76377291 was the most strongly associated with M-Index (*P* = 0.009; **S4 Fig**). Overall, the associations were heterogenous between the GWAS discovery and replication dataset.

**Table 2:** Association of 17 GWAS identified SNPs with skin pigmentation (M-Index).

					Discovery (n = 395)			Replication (n = 681)			Pooled (n = 1,076)	
CHR	SNP	BP	Gene	MA	MAF	*β*	*P*	MAF	*β*	*P*	*β*	*P*
2	rs79952417	22662120	*KLHL29*	G	0.03	-0.061	5.46 x 10^−6^	0.030	0.002	0.87	-0.023	0.009
4	rs12644472	42809090	*GRXCR1*	T	0.15	-0.028	8.96 x 10^−6^	0.132	-0.012	0.03	-0.018	2.08 x 10^−5^
4	rs13111738	176513040	*GPM6A*	T	0.30	0.025	1.28 x 10^−6^	0.310	0.002	0.66	0.010	0.003
5	rs2561059	91051911	*ARRDC3*	C	0.27	-0.024	7.32 x 10^−6^	0.251	0.007	0.10	-0.003	0.33
6	rs73424678	39402909	*KIF6*	G	0.13	-0.026	4.41 x 10^−6^	0.051	-0.005	0.29	-0.009	0.06
6	rs672706	164473619	*QKI*	C	0.20	0.028	4.02 x 10^−6^	0.236	0.004	0.65	0.005	0.21
7	rs9648318	25466032	*NPVF*	C	0.46	0.023	3.50 x 10^−6^	0.482	0.002	0.84	0.028	0.004
7	rs116746926	41651338	*INHBA*	G	0.03	0.071	1.66 x 10^−6^	0.024	0.002	0.69	0.007	0.02
10	rs3004256	43465912	*RET*	G	0.05	-0.053	8.64 x 10^−6^	0.053	0.020	0.02	-0.001	0.92
12	rs12370471	73038439	*TRHDE*	G	0.09	-0.046	1.28 x 10^−6^	0.085	-0.002	0.78	-0.016	0.005
12	rs11179301	73041679	*TRHDE*	T	0.08	-0.048	6.28 x 10^−7^	0.089	0.000	0.98	-0.016	0.005
12	rs11179306	73047637	*TRHDE*	A	0.08	-0.048	1.18 x 10^−6^	0.072	-0.002	0.75	-0.016	0.008
13	rs74377764	81516453	*SPRY2*	G	0.10	0.039	7.90 x 10^−6^	0.117	0.000	0.97	0.010	0.04
15	rs2675345	48400199	*SLC24A5*	A	0.20	-0.047	8.38 x 10^−14^	0.234	-0.039	5.45 x 10^−18^	-0.042	4.04 x 10^−30^
16	rs80009450	86366702	*FOXF1*	T	0.08	-0.042	5.41 x 10^−6^	0.065	0.004	0.66	-0.012	0.06
18	rs28802380	1859607	*METTL4*	A	0.27	0.024	7.49 x 10^−6^	0.252	-0.003	0.56	-0.015	0.0006
18	rs10503107	63946605	*CDH19*	A	0.15	-0.033	1.73 x 10^−6^	0.163	-0.003	0.54	0.007	0.04

CHR (Chromosome), BP (Basepair Position in GRCh37), MA (minor allele), MAF (minor allele frequency). Genes closest to the identified SNPs are listed

We also performed a replication analysis of 24 previously identified variants associated with pigmentation traits in European and admixed populations with our combined GWAS and replication dataset. Fifteen SNPs in 10 regions were replicated with *P*<0.05 and SNPs from 3 regions reached genome-wide significance (**Table 3**). A SNP, rs2470102, on *SLC24A5* showed the strongest association with M-Index (*P* = 9.86 x 10^−30^). The second strongest association was observed for a *SLC45A2* variant, rs16891982 (1.93 x 10^−13^). A SNP, rs1800404 in the *OCA2* gene was also strongly associated with M-Index (*P* = 4.94 x 10^−8^).

**Table 3:** Results of replication analysis of 24 previously identified pigmentation traits SNPs.

CHR	SNP	BP	Gene	MA	MAF	*β*	*P*	Reference
5	rs16891982	33951693	*SLC45A2*	G	0.18	-0.029	1.93 x 10^−13^	[2]
5	rs26722	33963870	*SLC45A2*	T	0.05	0.017	0.01	[77]
6	rs12203592	396321	*IRF4*	T	0.04	-0.018	0.02	[5]
6	rs262825	158678631	*GTF2H5*,*TULP4*	G	0.37	0.004	0.19	[54]
7	rs702477	12660526	*SCIN*	C	0.31	0.000	0.89	[54]
7	rs12668421	55109177	*EGFR*	T	0.09	-0.001	0.89	[78]
9	rs13289810	12396731	*TYRP1*	G	0.24	-0.005	0.13	[79]
9	rs1408799	12672097	*TYRP1*	C	0.28	-0.004	0.20	[3]
9	rs2733832	12704725	*TYRP1*	T	0.15	-0.011	0.01	[2, 80]
11	rs35264875	68846399	*TPCN2*	T	0.03	-0.025	0.005	[3]
11	rs3829241	68855363	*TPCN2*	A	0.11	-0.010	0.04	[3]
11	rs10831496	88557991	*GRM5*,*TYR*	A	0.23	-0.010	0.004	[4]
11	rs1042602	88911696	*TYR*	A	0.08	-0.020	0.0003	[2, 35, 55]
12	rs12821256	89328335	*KITLG*	C	0.04	0.005	0.47	[55]
14	rs12896399	92773663	*SLC24A4*	T	0.09	-0.002	0.63	[55]
15	rs1800404	28235773	*OCA2*	T	0.22	-0.020	4.94 x 10^−8^	[35, 81]
15	rs12913832	28365618	*OCA2/HERC2*	G	0.14	-0.023	2.52 x 10^−7^	[5]
15	rs1426654	48426484	*SLC24A5*	A	0.22	-0.041	2.36 x 10^−29^	[51]
15	rs2470102	48433494	*SLC24A5*	A	0.22	-0.042	9.86 x 10^−30^	[53]
16	rs1805007	89986117	*MC1R*	T	0.01	-0.027	0.03	[55]
16	rs2228478	89986608	*MC1R*	G	0.43	0.007	0.03	[35]
20	rs4911414	32729444	*ASIP*	T	0.15	0.001	0.86	[3]
20	rs6058017	32856998	*ASIP*	A	0.33	-0.008	0.01	[3, 29]
21	rs2835621	38510616	*TTC3-DSCR9*	G	0.36	-0.005	0.09	[82]

CHR (Chromosome), BP (Base Pair Position), MA (minor allele), MAF (minor allele frequency). Genotyping using MassARRAY was performed for the samples initially genotyped with Affymetrix platform, resulting in a total of 1,066 participants in this analysis after the QC procedures.

### Association of Genetic Score with skin pigmentation and sex interaction

To examine how strongly the identified pigmentation gene variants combined were associated with skin pigmentation, a weighted Genetic Score was calculated with the top SNPs of the 10 most significantly associated loci as well as the top SNPs from 3 loci that reached genome-wide significance (**S1 Table**).

**Table 4** shows the association between the Genetic Scores and M-Index. The Genetic Scores were positively associated with M-Index (*P*<0.001). Age and sex explained a very small proportion of variance in M-Index (0.3% and 1.7% respectively). WAA explained a large proportion of variance (23.2%). When the Genetic Score was added to the regression model with age, sex, and WAA, the Genetic Score from the top 3 SNPs explained additional 11% of skin pigmentation variance. Using the Genetic Score from the top 10 SNPs to the regression model instead of Genetic Score from 3 SNPs yielded a similar result, and the top 10 SNPs accounted for 12% of variance. In our subsequent analysis, we used the Genetic Score from the top 3 SNPs including rs2470102 (*SLC24A5*), rs16891982 (*SLC45A2*), and rs1800404 (*OCA2*).

**Table 4:** Genetic Scores and association with M-Index.

	10 SNPs^1^			3 SNPs^2^		
	*R*^*2*^	*β*	*P*	*R*^*2*^	*β*	*P*
Whole Model	0.372			0.362		
Age	0.003	-0.001	<0.001			
Sex (Females)	0.017	-0.013	0.07			
WAA	0.232	0.166	<0.001			
Genetic Score	0.120	0.687	<0.001	0.110	0.801	<0.001
Among Males		0.849	<0.001		0.895	<0.001
Among Females		0.316	0.001		0.497	<0.001
Sex x Genetic Score Interaction			<0.001			0.009

^1^ 10 SNPs independently associated SNPs were included (Supplementary Table 2). A total of 1,012 samples with complete genotype data for 10 SNPs were included.

^2^ Genetic Score was calculated using top three SNPs, rs2470102 (*SLC24A5*), rs16891982 (*SLC45A2*), and rs1800404 (*OCA2*) that reached genome wide significance. A total of 1,062 samples with complete genotype data for the 3 SNPs were included.

**Fig 2:**
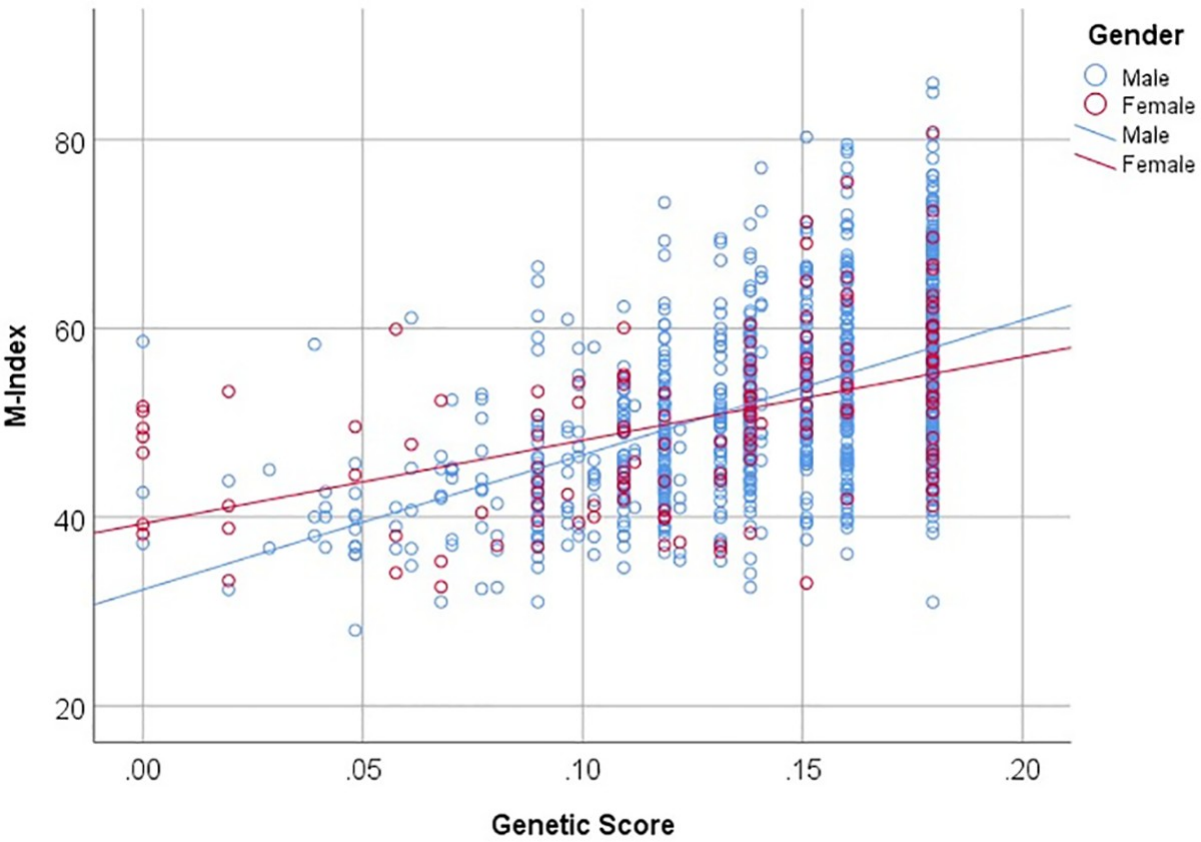
Sex and Genetic Score interaction. The Genetic Score was calculated using 3 independent loci associated with M-Index.

Although both the Genetic Score and WAA were strongly associated with M-Index, high variance in M-Index was observed among the study participants with the highest Genetic Score and WAA (**S5 Fig)**. When the association between the Genetic Score and M-Index was examined separately for male and female study participants, a stronger genetic effect was observed in male than female study participants (**Table 4** and **Fig 2**). The interaction between sex and Genetic Score was statistically significant (*P* = 0.009).

Because we had an unbalanced representation of male and female study participants with a small sample size for females, sex-specific Genetic Scores were calculated to further assess the associations between Genetic Scores and M-Index. The top 3 SNPs included for calculation of sex-specific Genetic Scores were associated with M-Index in both males and females, when sex stratified analysis was performed (**S2 Table**). The results of sex-specific Genetic Scores and stratified analysis based on sex using overall Genetic Score were similar (**S3 Table**). The association between sex-specific Genetic Score and M-Index was stronger for males than for females, and males had a larger *R*^*2*^ and *β* estimate than females.

### Association between skin pigmentation gene variants and vitamin D deficiency

Next, we assessed the role of skin pigmentation in serum 25(OH)D levels among male study participants who were part of vitamin D and prostate cancer studies. The M-Index was not associated with serum 25(OH)D levels, but the Genetic Score for skin pigmentation was significantly negatively associated with serum 25(OH)D levels (*P* = 0.01; **S4 Table**). The Genetic Score calculated with the top 3 skin pigmentation gene SNPs accounted for a small proportion of serum 25(OH)D variance (0.7%). WAA estimates were not significantly correlated with serum 25(OH)D levels (Spearman’s *ρ* = 0.015, *P* = 0.68), and the association was not significant in the linear regression model. When we examined the association between the top 10 SNPs associated with skin pigmentation (**S1 Table**) and serum 25(OH)D levels, two SNPs, rs2470102 on *SLC24A5* (*P* = 0.004) and rs2733832 on *TYRP1* (*P* = 0.04) were significantly associated with serum 25(OH)D levels (**S5 Table**).

Genotype data for 38 SNPs in 8 vitamin D metabolic and signaling pathway genes were also available for these individuals, and 3 SNPs were associated with serum 25(OH)D levels in this dataset (rs1155563 in *GC*, rs12800438 in *DHCR7/NADSYN1*, and rs11574143 in *VDR*, **S6 Table**). A Genetic Score for serum 25(OH)D levels was calculated using these 3 SNPs. The Genetic Score was strongly associated with serum 25(OH)D levels (*P*<0.001), but these 3 SNPs explained only 1.9% of variance. Genetic Scores using 3 skin pigmentation gene SNPs and 3 vitamin D metabolic and signaling gene SNPs together explained 2.9% of serum 25(OH)D variance, when they were included in the regression model together.

Finally, we examined how strongly the top 3 skin pigmentation gene SNPs are associated with vitamin D deficiency (**Table 5**). Increasing the number of alleles associated with darker pigmentation (when Genetic Score was treated as an ordinal variable) significantly increased the odds of having severe vitamin D deficiency (OR, 1.34; 95% CI, 1.02–1.86). The odds of severe vitamin D deficiency was even larger when Genetic Score Tertile 1 was compared to Tertile 3 (OR, 1.83; 95% CI, 1.02–3.29). Additional analysis was performed using the male-specific Genetic Score. Overall and male-specific Genetic Scores were highly correlated, and the analysis with male-specific Genetic Score produced identical results with overall Genetic Score.

**Table 5:** Top three skin pigmentation SNPs are associated with severe vitamin D deficiency in African American men.

	<12 ng/mL vs. ≥12 ng/mL	
	OR (95% C.I.)	*P*_TREND_
Tertiles from Genetics Score (ordinal)	1.34 (1.02–1.86)	0.03
Tertiles from Genetic Score		
Tertile 1	Reference	
Tertile 2	0.97 (0.56–1.69)	
Tertile 3	1.83 (1.02–3.29)	

*P*-value for linear trend was estimated treating tertiles of Genetic Score as an ordinal variable. Genetic Score was calculated using three SNPs, rs2470102 (*SLC24A5*), rs16891982 (*SLC45A2*), and rs1800404 (*OCA2*). A total of 591 men who had all the covariates and genotype information for 3 skin pigmentation genes and 3 vitamin D metabolic and signaling pathway genes were included.

## Discussion

In this first GWAS of skin pigmentation in AAs, we demonstrated that variants near the *SLC24A5* gene on chromosome 15 revealed the strongest signal of association in AAs, supporting findings from previous studies in African populations [8,9] and admixed populations [2,7,10]. A variant from this locus along with a variant from both *SLC45A2* and *OCA2* together accounted for most of the identified genetic variance in M-Index variance (**Table 4**). However, WAA accounted for a greater proportion of skin pigmentation variance, and we observed great variation in M-Index among AAs with high WAA and genetic score, suggesting that other unknown genomic factors related to WAA are likely contributing to skin pigmentation variation. We also demonstrated that skin pigmentation gene variants were associated with serum 25(OH)D levels in AAs providing evidence to support a role of skin pigmentation in serum 25(OH)D variation.

The top 3 regions that were associated with skin pigmentation were *SLC24A5*, *SLC45A2*, and *OCA2*, and variants in these genes explained a large proportion of skin pigmentation variation in our dataset. The variants in the *SLC24A5* are the most strongly associated with skin pigmentation in African descent populations and the associations between them has been demonstrated in AAs [51,52], Cape Verde population [53], and African populations [8,9] as well as meta-analysis in admixed populations [10], but not in European populations [5,54]. The variants in *SLC45A2* and *OCA2* region also showed very strong associations with skin pigmentation in the African-European admixed population from Cape Verde [53]. Strong associations between variants in these two genes and skin pigmentation or other pigmentation traits have also been shown in European population [1,5,55]. However, African populations appear to have a more complex genetic architecture for skin pigmentation. Crawford and colleagues showed variants in *OCA2* and *HERC2* region as the fourth most significantly associated locus, but *SLC45A2* region was not one of the most significantly associated loci. In the KhoeSan populations from southern Africa, variants in these two regions did not show strong association with skin pigmentation. Instead, these two studies in African populations identified novel loci in *SNX13*, *SMARCA2/VLDLR*, *TMEM138/DDB1*, and *MFSD12* associated with skin pigmentation. The associations between *MFSD12* variants and skin pigmentation were validated in Latin American populations [10] and meta-analysis of recently admixed populations [10]. These loci may explain additional skin pigmentation variance captured by WAA, but unexplained by the Genetic Score. Because the index SNP from each locus in these studies was not successfully imputed in our GWAS, we looked for SNPs associated with skin pigmentation in these regions. However, variants in these regions were not strongly associated with skin pigmentation. It is likely that variants in these regions contribute to skin pigmentation variation with moderate effect, and a larger sample size is necessary to replicate these findings. Furthermore, SNPs in *IRF4*, *TPCN2*, *TYR*, and *MC1R* associated with pigmentation traits in European populations were replicated [3–5,55], but these variants contribute a small proportion of skin pigmentation variation in AAs.

We identified a potentially novel locus on the *TRHDE* gene associated with skin pigmentation. The gene, *TRHDE* (thyrotropin releasing hormone degrading enzyme), encodes an enzyme that specifically cleaves and inactivates thyrotropin-releasing hormone (TRH). The TRH is produced in the hypothalamus and stimulates production of thyroid-stimulating hormone (TSH). A SNP, rs2044305, in *TRHDE* has been identified as a candidate variant influencing TSH levels [56]. Thyroid-stimulating hormone as well as melanocyte-stimulating hormone are produced in the anterior pituitary gland. TRH also stimulates growth hormone, prolactin, and α-melanocyte-stimulating hormone in fish, amphibians, and mammals, and these pituitary hormones play important roles in skin [57]. TRH also stimulate melanin synthesis in human hair follicle, potentially by binding to the melanocortin-1 receptor (MC1-R) [58,59]. It is interesting to note that horses with pituitary pars intermedia dysfunction have abnormal coat sometime with lighter color and elevated plasma α-melanocyte-stimulating hormone levels. Administering TRH to the healthy horses increases plasma α-melanocyte-stimulating hormone concentrations [60]. Polymorphisms in the *TRHDE* gene may alter TRH levels, and subsequently α-melanocyte-stimulating hormone production in the pituitary grand. We selected the top 3 SNPs in the *TRHDE* region for replication analysis. However, associations of these SNPs were not replicated in the independent dataset. Because of the strong suggestive association in GWAS dataset and a potential biological importance, additional *TRHDE* SNPs were genotyped to further explore the association with skin pigmentation. The associations of *TRHDE* SNPs with skin pigmentation were heterogeneous between discovery and replication dataset, but we were successful in replication at a gene level with a different SNP, rs76377291 showing a strong association with skin pigmentation in replication dataset. The SNP rs76377291 is located about 28kb from rs11179301, the lead SNP in the region in our GWAS and these two SNPs were not strongly correlated in 1000 Genome Project ASW population (*r*^*2*^ = 0.05, *D’* = 1.0). It is possible that functional variants exist in a nearby location, and we did not capture that genomic variation in our replication data set. Further fine-mapping may help identify the functional variants within this region.

This study explored the associations between skin pigmentation gene variants and serum vitamin D levels in AAs. It has been hypothesized that human skin de-pigmentation evolved as our early ancestors migrated into low UV environment with increased needs for subcutaneous vitamin D synthesis [11] and numerous medical conditions associated with vitamin D deficiency, such as bone disorders, susceptibility to infections, autoimmune disorders, cancer, and reproductive health suggest importance of vitamin D in human health [61]. Despite the important role that skin pigmentation plays for vitamin D status, a few studies examined the relationship between skin pigmentation and vitamin D status in AAs. Studies have shown that individuals with darker skin pigmentation require longer and/or more intense UVR exposure to synthesize sufficient levels of vitamin D [62–64]. Genetic ancestry estimates were also associated with serum 25(OH)D levels in AAs in Southern Community Cohort Study and Black Women’s Health Study [65,66]. These observations led many to believe that disparities in vitamin D status between AAs and EAs is partly due to difference in skin pigmentation [65–67].

Contrary to these studies, genetic ancestry was not associated with serum 25(OH)D levels in our study of AA men. Location of residence, sex, and other factors affect serum 25(OH)D levels, and the differences between our study and previous studies may explain the inconsistent finding. Moreover, there has been no study which explored the association between skin color variation and serum vitamin D levels in AAs using objective measurements. In this study, skin pigmentation gene variants rather than skin pigmentation measured using a reflectometer were associated with serum vitamin D levels. Although skin pigmentation was measured in an area of the body unexposed to the sun, various factors, such as aging, outdoor activities, and consistent UV exposure over the years, may influence skin pigmentation and the association between skin pigmentation and serum vitamin D levels. Because genotype is assigned randomly at conception, the association between skin pigmentation gene variants and serum vitamin D levels is unlikely to be affected by confounding factors [68]. Pigmentation Genetic Scores were also associated with serum 25(OH)D levels in previous studies among children and adult men in the United Kingdom [69,70]. However, the contribution of skin pigmentation genomic variation to serum vitamin D variance was small in our study as well as previous studies. The relationship between skin pigmentation gene variants and serum vitamin D levels should be further examined.

There are some limitations of this study. First, this study had a small sample size for the initial GWAS discovery sample set (n = 395). Thus, we were only able to show association of variants with strong effects. It is likely there are many other yet unknown variants with small effects that influence skin pigmentation in African descent populations. SNPs with *P*-value less than 1.0 x 10^−5^ were included in the replication analysis to identify novel variants associated with skin pigmentation with smaller effect size in AAs. One SNP on chromosome 4, rs12644472, was replicated, but did not reach genome-wide significance in the pooled analysis. This SNP is located between two genes, *GRXCR1* (glutaredoxin and cysteine rich domain containing 1) and *ATP8A1* (ATPase phospholipid transporting 8A1). Mutations in the *GRXCR1* genes are linked to hearing loss [71,72]. A GWAS of saggy eyelids showed suggestive associations of *ATP8A1* SNPs in European populations, suggesting potential roles of *ATP8A1* in skin [73].

Second, a less explored area of pigmentation research is genetic and sex effects on skin pigmentation [74,75]. A previous study found evidence of interaction between *ASIP* genotypes and sex in AAs [29]. Instead of using genotype, we calculated overall and sex-specific Genetic Scores and demonstrated that the strength of associations between the Genetic Scores and skin pigmentation were different between men and women. Sex hormones may modify genetic effects on skin pigmentation [76]. However, we had an over-representation of men in this study, because many study participants were recruited to prostate cancer risk studies. This observation should be validated incorporating much larger samples of AA women.

Finally, it is likely that the calculated Genetic Scores were over-fitted, because SNPs used for calculation of Genetics Score were selected based on our results instead of using a previously verified set of genetic markers for skin pigmentation and serum vitamin D levels in AA populations. Currently, there is no comprehensive genomic study of skin pigmentation and serum vitamin D levels in AA populations.

In conclusion, our results show strong associations between polymorphisms in 3 major pigmentation genes and skin pigmentation in AAs. The variants in these genes explained a large proportion of skin pigmentation variation and the effects of these variants on skin pigmentation was modified by sex. We also demonstrated that skin pigmentation gene variants were associated with serum vitamin D levels providing support for the vitamin D hypothesis of skin color evolution.

## Supporting information

S1 Table 10 SNPs used for calculation of Genetic Score in GWAS and replication combined dataset (n = 1,066). (PDF)

S2 Table Top 3 SNPs associated with M-Index used for calculation of sex-specific Genetic Scores in GWAS and replication combined dataset (n = 1,066). (PDF)

S3 Table Associations between sex-specific Genetic Scores and M-Index. (PDF)

S4 Table Associations between Genetic Scores (from 3 pigmentation gene SNPs and 3 vitamin D pathway gene SNPs) and serum vitamin D levels. (PDF)

S5 Table Association between 10 skin pigmentation SNPs and serum vitamin D levels. (PDF)

S6 Table Variants in Vitamin D metabolic and signaling pathway gene variants associated with serum 25(OH)D levels (n = 606). (PDF)

S1 Fig QQ plot indicating there was no evidence of genomic inflation (Inflation factor λ = 1.028) using first three PCs. (PDF)

S2 Fig Association between *TRHDE* SNPs on chromosome 12 and M-Index. (PDF)

S3 Fig LocusZoom Plots of 4 Genomic Regions Identified in GWAS of African Populations. (PDF)

S4 Fig Heterogeneous associations between *THRD* variants and M-Index in replication and GWAS dataset. (PDF)

S5 Fig Correlation between M-Index and Genetic Score (A), between M-Index and West African Ancestry (WAA) (B), and between Genetic Score and WAA (C). Correlations were significant with Spearman’s correlation *P*<0.001.
(PDF)
