# Supplementary material for: Genetic loci associated with skin pigmentation in African Americans and their effects on vitamin D deficiency
Source: PLoS Genet. 2021 Feb 18;17(2):e1009319. doi: 10.1371/journal.pgen.1009319 (PMC7891745; doi:10.1371/journal.pgen.1009319)
Supplement: S1 Table — (PDF) [file pgen.1009319.s001.pdf]

S1 Table 10 SNPs used for calculation of Genetic Score in GWAS and replication combined dataset (n=1,066). SNPs were selected from Table 3. SNP with the lowest *P*-value from each genomic region was included.

				MAF (%)				
CHR	SNP	BP	MA	EUR	AFR	*β*	R^2	*P*
15	rs2470102	48433494	A	0.99	0.01	-0.042	0.096	9.86×10^{-30}
5	rs16891982	33951693	G	0.94	0.04	-0.029	0.045	1.93×10^{-13}
15	rs1800404	28235773	T	0.79	0.07	-0.020	0.030	4.94×10^{-8}
11	rs1042602	88911696	A	0.37	0.01	-0.020	0.019	0.0003
4	rs12644472	42809090	T	0.13	0.12	-0.016	0.017	0.0004
11	rs35264875	68846399	T	0.20	0.02	-0.025	0.015	0.005
6	rs12203592	396321	T	0.12	0.01	-0.018	0.014	0.02
9	rs2733832	12704725	T	0.57	0.04	-0.011	0.014	0.01
20	rs6058017	32856998	A	0.90	0.21	-0.008	0.013	0.01
16	rs1805007	89986117	T	0.07	0.01	-0.027	0.013	0.03

CHR (Chromosome), BP (Basepair Position), MA (minor allele), MAF (minor allele frequency)
