# Supplementary material for: Genetic loci associated with skin pigmentation in African Americans and their effects on vitamin D deficiency
Source: PLoS Genet. 2021 Feb 18;17(2):e1009319. doi: 10.1371/journal.pgen.1009319 (PMC7891745; doi:10.1371/journal.pgen.1009319)
Supplement: S2 Table — (PDF) [file pgen.1009319.s002.pdf]

S2 Table Top 3 SNPs associated with M-Index used for calculation of sex-specific Genetic Scores in GWAS and replication combined dataset (n=1,066).

CHR	SNP	BP	MA	MAF	*β*	R^2	*P*
Males (n=932)							
15	rs2470102	48433494	A	0.20	-0.0438	0.089	2.06×10^{-26}
5	rs16891982	33951693	G	0.17	-0.0302	0.035	7.72×10^{-12}
15	rs1800404	28235773	T	0.21	-0.018	0.017	6.83×10^{-6}
Females (n=132)							
15	rs2470102	48433494	A	0.33	-0.0306	0.070	0.0001
15	rs1800404	28235773	T	0.27	-0.0239	0.041	0.003
5	rs16891982	33951693	G	0.27	-0.0201	0.027	0.02
