# Supplementary material for: Genetic loci associated with skin pigmentation in African Americans and their effects on vitamin D deficiency
Source: PLoS Genet. 2021 Feb 18;17(2):e1009319. doi: 10.1371/journal.pgen.1009319 (PMC7891745; doi:10.1371/journal.pgen.1009319)
Supplement: S3 Table — (PDF) [file pgen.1009319.s003.pdf]

S3 Table Associations between sex-specific Genetic Scores and M-Index.

	Males			Females		
	R^2	β	P	R^2	β	P
Whole Model	0.367			0.448		
Age	0.008	-0.001	<0.001	0.074	-0.001	0.01
WAA	0.233	0.175	<0.001	0.292	0.216	<0.001
Genetic Score[1]	0.126	0.892	<0.001	0.082	0.607	<0.001

Weighted sex-specific Genetic Score was calculated using top three SNPs, rs2470102 (*SLC24A5*), rs16891982 (*SLC45A2*), and rs1800404 (*OCA2*) associated with M-Index.
