# Supplementary material for: Genetic loci associated with skin pigmentation in African Americans and their effects on vitamin D deficiency
Source: PLoS Genet. 2021 Feb 18;17(2):e1009319. doi: 10.1371/journal.pgen.1009319 (PMC7891745; doi:10.1371/journal.pgen.1009319)
Supplement: S4 Table — (PDF) [file pgen.1009319.s004.pdf]

S4 Table Associations between Genetic Scores (from 3 pigmentation gene SNPs and 3 vitamin D pathway gene SNPs) and serum vitamin D levels.

	R^2	β	P
Base Model[1]	0.179		
+ M-Index[2]	0.001	-0.038	0.34
+ Genetic Score (Pigmentation SNPs)[2]	0.007	-0.099	0.01
+ Genetic Score (Vitamin D Pathway SNPs)[2]	0.019	0.139	<0.001
+ Genetic Score (Pigmentation) + Genetic Score (Vitamin D)[3]	0.029		
Genetic Score (Pigmentation SNPs)		-0.116	0.007
Genetic Score (Vitamin D SNPs)		0.136	<0.001

[1] Base model includes age, WAA, UV season (Season of blood draw), study site, and total vitamin D intake.

[2] Subsequently, M-Index, Genetic Score estimated from 3 pigmentation gene SNPs, or Genetic Score from 3 vitamin D pathway gene SNPs were added to estimate the variance explained by M-Index or Genetic Scores. Genetic Score of vitamin D metabolic pathway genes were calculated using rs1155563 (*GC*), rs12801438 (*DHCR7*/*NADSYN1*), and rs11574143 (*VDR*). Genetic Score of pigmentation genes was calculated using rs2470102 (*SLC24A5*), rs16891982 (*SLC45A2*), and rs1800404 (*OCA2*).

[3] Both Genetic Scores (from pigmentation gene SNPs and vitamin D pathway gene SNPs) were added to the base model to estimate the variance explained by both Genetic Scores together.
