# Supplementary material for: Genetic loci associated with skin pigmentation in African Americans and their effects on vitamin D deficiency
Source: PLoS Genet. 2021 Feb 18;17(2):e1009319. doi: 10.1371/journal.pgen.1009319 (PMC7891745; doi:10.1371/journal.pgen.1009319)
Supplement: S5 Table — (PDF) [file pgen.1009319.s005.pdf]

S5 Table Association between 10 skin pigmentation SNPs and serum vitamin D levels

CHR	SNP	BP	MA	*β*	R^2	*P*
15	rs2470102	48433494	A	-0.108	0.010	**0.004**
5	rs16891982	33951693	G	0.009	0.000	0.82
15	rs1800404	28235773	T	-0.034	0.001	0.34
11	rs1042602	88911696	A	0.022	0.000	0.54
4	rs12644472	42809090	T	0.023	0.001	0.50
11	rs35264875	68846399	T	0.014	0.000	0.69
6	rs12203592	396321	T	-0.009	0.000	0.80
9	rs2733832	12704725	T	-0.059	0.005	**0.04**
20	rs6058017	32856998	A	-0.032	0.001	0.35
16	rs1805007	89986117	T	-0.028	0.001	0.42

Adjusting for age, WAA, UV season (season of blood draw), study site, and total vitamin D intake
