# Supplementary material for: Genetic loci associated with skin pigmentation in African Americans and their effects on vitamin D deficiency
Source: PLoS Genet. 2021 Feb 18;17(2):e1009319. doi: 10.1371/journal.pgen.1009319 (PMC7891745; doi:10.1371/journal.pgen.1009319)
Supplement: S6 Table — (PDF) [file pgen.1009319.s006.pdf]

S6 Table Variants in Vitamin D metabolic and signaling pathway genes associated with serum 25(OH)D levels (n=606).

CHR	SNP	BP	Gene	MA	β	P
2	rs116071925	219354945	*CYP27A1*	A	-0.047	0.31
4	rs115316390	72870023	*GC*	A	0.086	0.22
4	rs17467825	72970552		G	-0.031	0.12
4	rs2282679	72973418		C	-0.031	0.14
4	rs3755967	72974433		A	-0.021	0.31
4	rs2298850	72979302		G	-0.033	0.13
4	rs7041	72983369		G	0.012	0.42
4	rs1155563	73008523		C	-0.052	**0.02**
4	rs3733359	73014809		T	-0.005	0.70
4	rs16847024	73015714		T	-0.016	0.49
7	rs2740574	99026747	*CYP3A4*	A	0.006	0.62
11	rs1993116	14866810	*CYP2R1*	T	0.020	0.15
11	rs12794714	14870151		A	-0.024	0.14
11	rs114050796	14871229		T	0.000	0.99
11	rs10741657	14871454		A	0.021	0.13
11	rs2060793	14871886		A	0.016	0.23
11	rs7944926	70843273	*DHCR7/NADSYN1*	G	-0.008	0.57
11	rs12785878	70845097		T	-0.006	0.65
11	rs4944957	70845683		A	-0.020	0.08
11	rs12800438	70848651		A	0.026	**0.04**
11	rs3794060	70865327		T	-0.001	0.94
11	rs3829251	70872207		A	-0.007	0.63
11	rs4945008	70898896		G	0.000	0.99
11	rs11234027	70911755		A	0.003	0.82
12	rs11574143	46521184	*VDR*	A	-0.043	**0.049**
12	rs731236	46525024		C	-0.007	0.64
12	rs1544410	46526102		A	-0.013	0.35
12	rs2228570	46559162		T	-0.007	0.63
12	rs11574038	46563420		A	-0.018	0.60
12	rs11568820	46588812		G	-0.025	0.10
12	rs4646537	56443548	*CYP27B1*	C	0.036	0.09
12	rs10877012	56448352		T	-0.011	0.55
20	rs6013897	52175886	*CYP24A1*	A	-0.008	0.58
20	rs6022990	52208939		G	-0.016	0.44
20	rs73913755	52223601		A	-0.015	0.36
20	rs73913757	52223925		T	-0.022	0.18
20	rs2248359	52224925		C	-0.001	0.90
20	rs2248461	52225609		G	0.003	0.82

Adjusted for age, WAA, UV season (season of blood draw), total vitamin D intake, and study site
