# Supplementary material for: Genetic loci associated with skin pigmentation in African Americans and their effects on vitamin D deficiency
Source: PLoS Genet. 2021 Feb 18;17(2):e1009319. doi: 10.1371/journal.pgen.1009319 (PMC7891745; doi:10.1371/journal.pgen.1009319)

S1 Fig QQ plot indicating there was no evidence of genomic inflation (Inflation factor λ=1.028) using first three PCs.

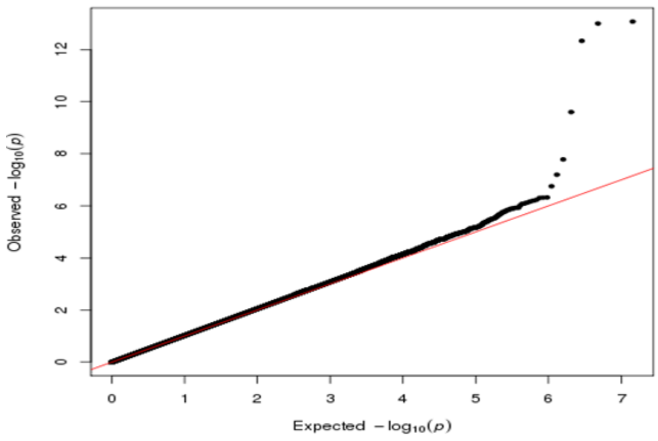

Supplement: S1 Fig — (PDF) [file pgen.1009319.s007.pdf]
