# Supplementary material for: Genetic loci associated with skin pigmentation in African Americans and their effects on vitamin D deficiency
Source: PLoS Genet. 2021 Feb 18;17(2):e1009319. doi: 10.1371/journal.pgen.1009319 (PMC7891745; doi:10.1371/journal.pgen.1009319)

S2 Fig Association between *TRHDE* SNPs on chromosome 12 and M-Index. LocusZoom plot of *TRHDE* region (A) and Box plot for *TRHDE* SNP rs11179301 (B). Mean M-Index for the rs11179301 genotype CC was 53.2 (±SD 10.2%) (Figure 1D). Only one individual had genotype TT, and mean M-Index for genotypes CT and TT combined was 48.5 (±SD 7.5%).

A.

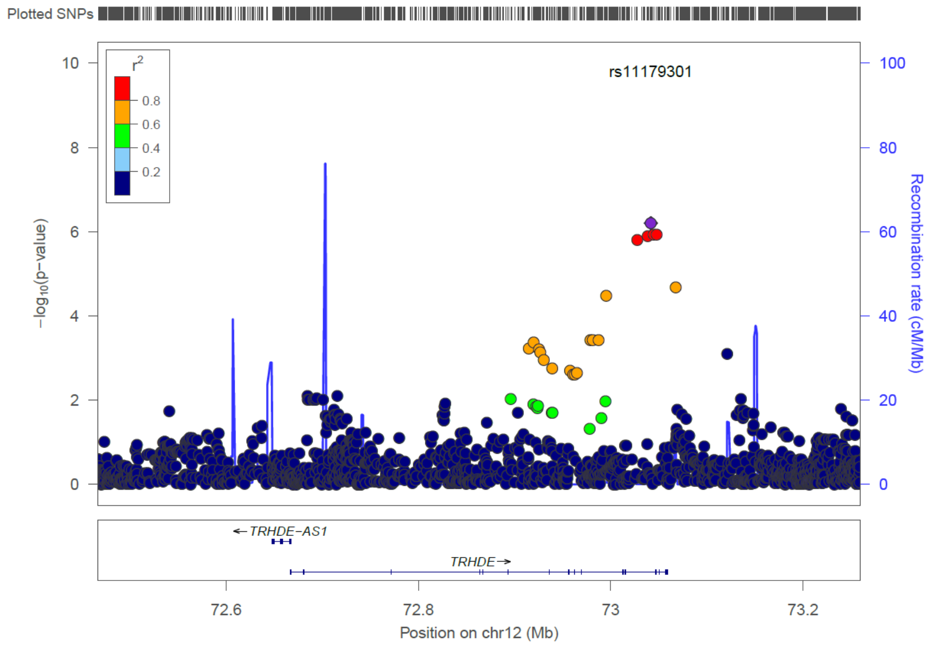

B.

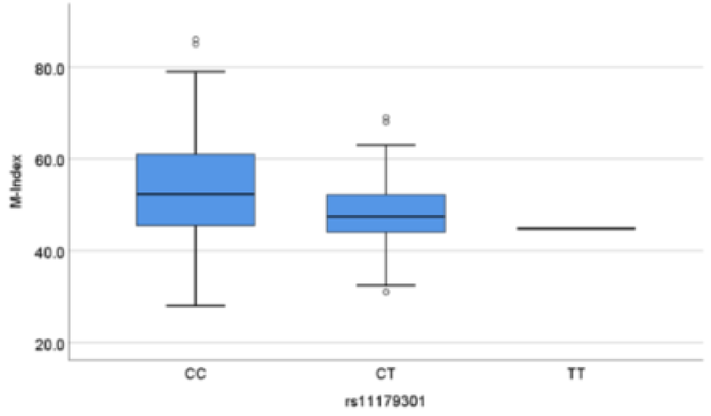

Supplement: S2 Fig — (PDF) [file pgen.1009319.s008.pdf]
