# Supplementary material for: Genetic loci associated with skin pigmentation in African Americans and their effects on vitamin D deficiency
Source: PLoS Genet. 2021 Feb 18;17(2):e1009319. doi: 10.1371/journal.pgen.1009319 (PMC7891745; doi:10.1371/journal.pgen.1009319)
Supplement: S3 Fig — (PDF) [file pgen.1009319.s009.pdf]

S3 Fig LocusZoom Plots of 4 Genomic Regions Previously Identified in GWAS of African Populations

SNX13

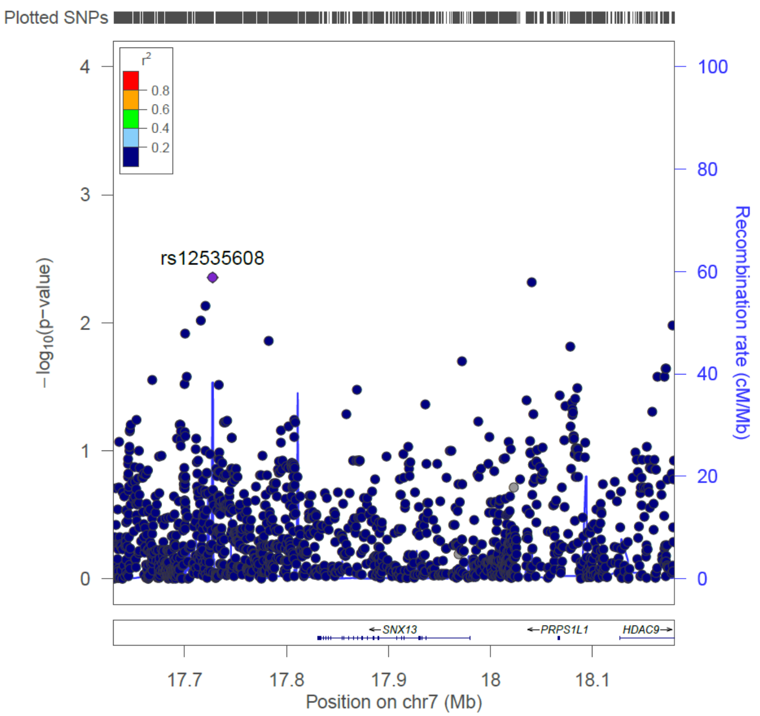

rs12535608 (P=4.4 x 10^{-3})

SMARCA2/VLDLR

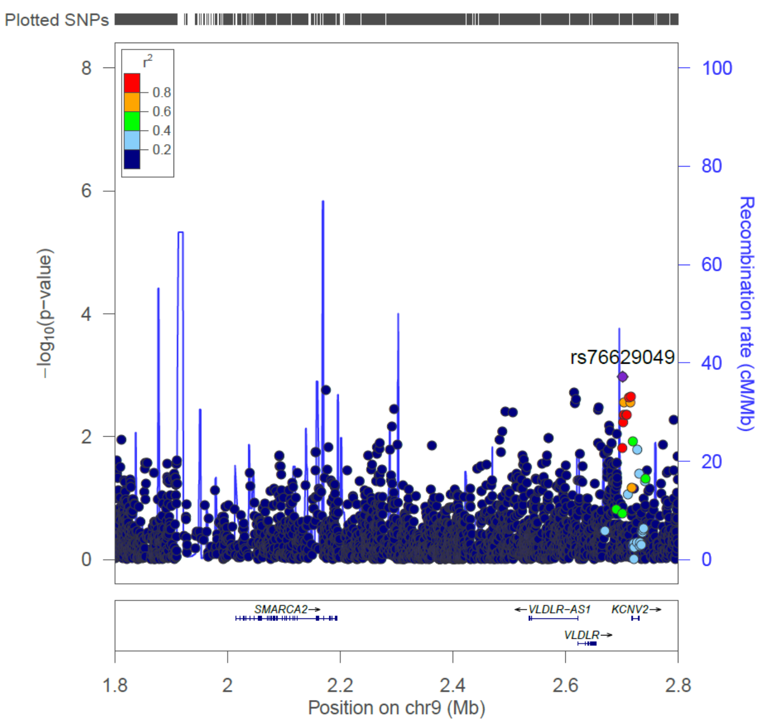

rs144734935 (P=1.7 x 10^{-3})

TMEM138/DDB1

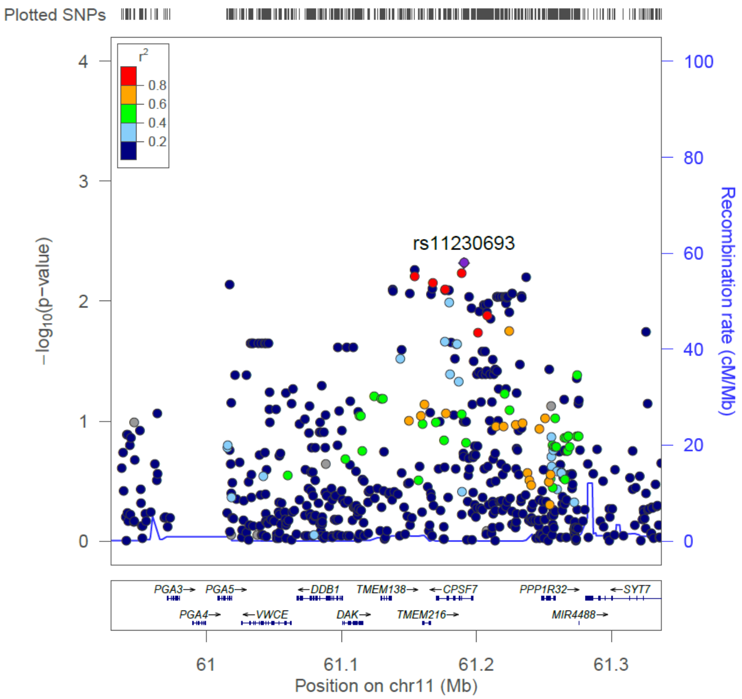

rs11230693 (P=4.8 x 10^{-3})

MFSD12

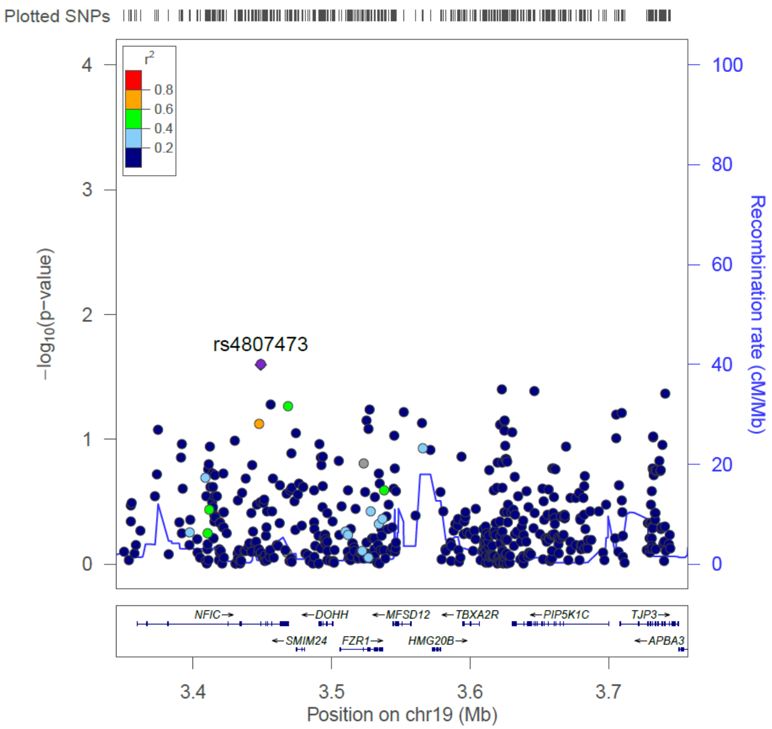

rs4807473 (P=0.03)
