# Supplementary material for: Genetic loci associated with skin pigmentation in African Americans and their effects on vitamin D deficiency
Source: PLoS Genet. 2021 Feb 18;17(2):e1009319. doi: 10.1371/journal.pgen.1009319 (PMC7891745; doi:10.1371/journal.pgen.1009319)

S4 Fig Heterogeneous associations between *THRDE* variants and M-Index in replication and GWAS dataset. Nine SNPs with minor allele frequency greater than 1% in the replication cohort were included for analysis. Three SNPs were associated with M-Index with *P*<0.05 in the replication dataset.

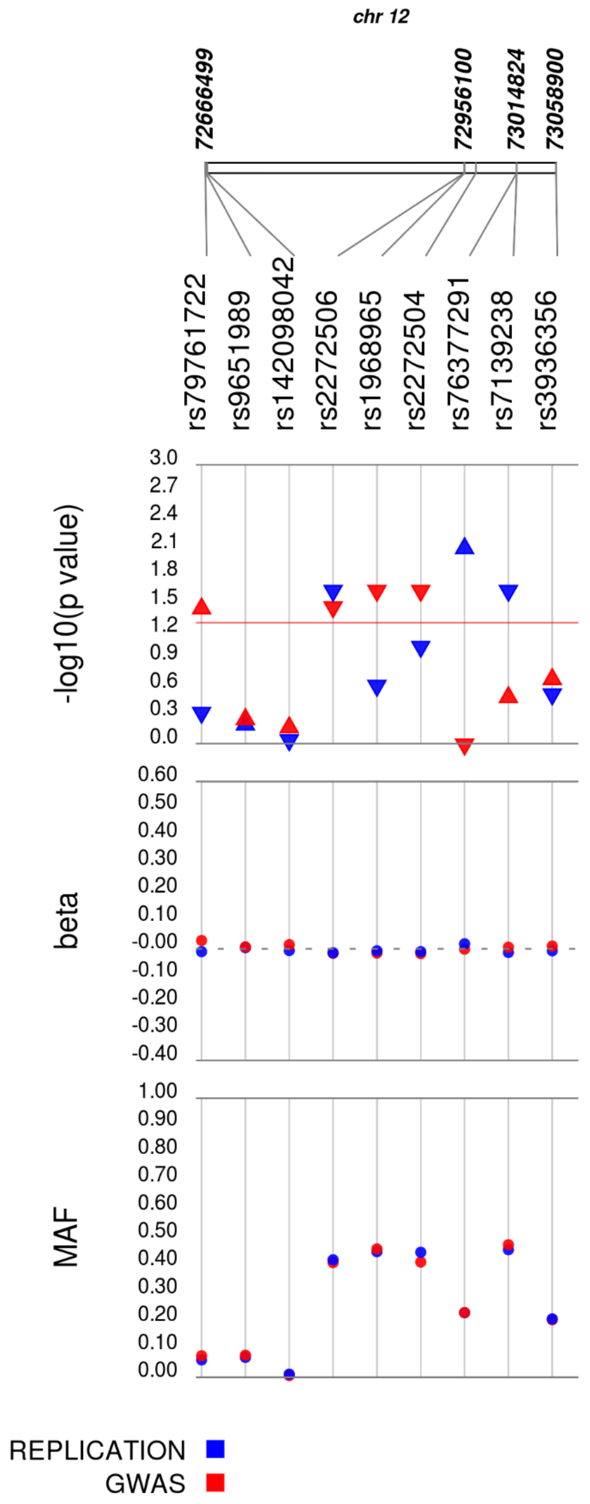

Supplement: S4 Fig — (PDF) [file pgen.1009319.s010.pdf]
