# Supplementary material for: Genetic loci associated with skin pigmentation in African Americans and their effects on vitamin D deficiency
Source: PLoS Genet. 2021 Feb 18;17(2):e1009319. doi: 10.1371/journal.pgen.1009319 (PMC7891745; doi:10.1371/journal.pgen.1009319)

S5 Fig Correlation between M-Index and Genetic Score (A), between M-Index and West African Ancestry (WAA) (B), and between Genetic Score and WAA (C). Correlations were significant with Spearman's correlation *P*<0.001.

A.

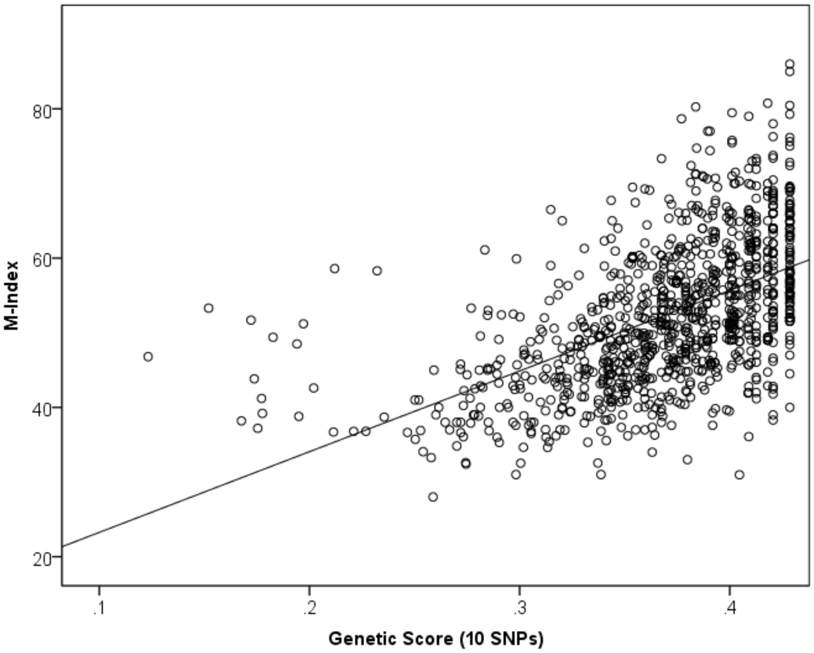

B.

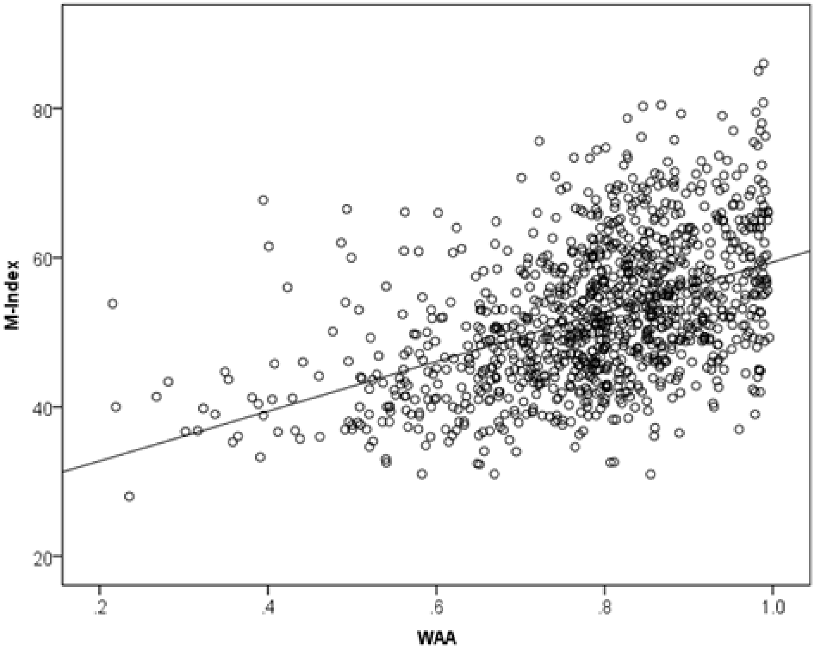

C.

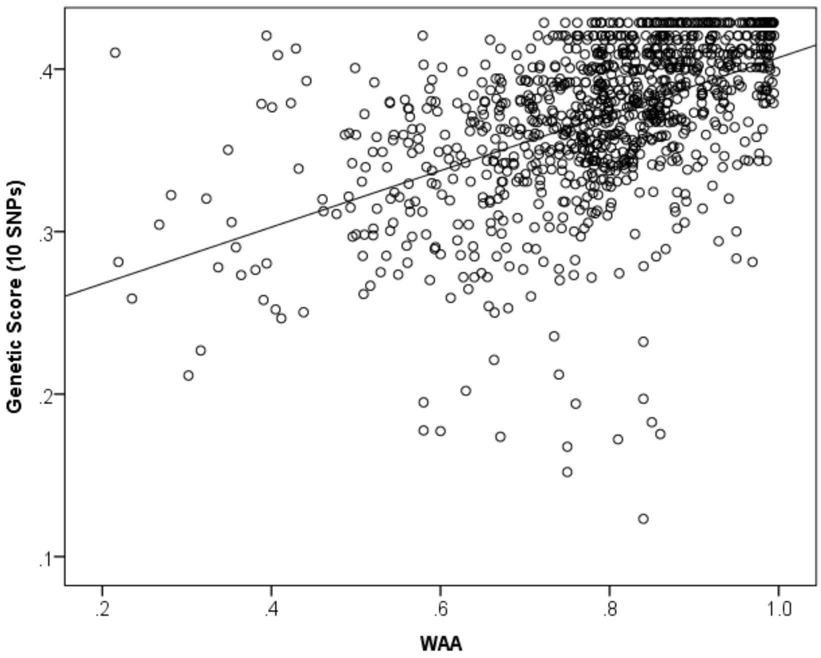

Supplement: S5 Fig — Correlation between M-Index and Genetic Score (A), between M-Index and West African Ancestry (WAA) (B), and between Genetic Score and WAA (C). Correlations were significant with Spearman’s correlation P<0.001. (PDF) [file pgen.1009319.s011.pdf]
